# Characteristics of Anterior Segment in Congenital Ectopia Lentis: An SS-OCT Study

**DOI:** 10.1155/2022/6128832

**Published:** 2022-06-06

**Authors:** Haotian Qi, Guangming Jin, Minjie Zou, Charlotte Young, Liyan Liu, Zhangkai Lian, Dongwei Guo, Zhenzhen Liu, Danying Zheng

**Affiliations:** ^1^State Key Laboratory of Ophthalmology, Zhongshan Ophthalmic Center, Sun Yat-sen University, Guangdong Provincial Key Laboratory of Ophthalmology and Visual Science, Guangdong Provincial Clinical Research Center for Ocular Diseases, Guangzhou 510060, China; ^2^Department of Ophthalmology, Third Affiliated Hospital, Nanchang University, Nanchang, Jiangxi Province, China

## Abstract

**Purpose:**

To investigate the characteristics of anterior chamber angle parameters in congenital ectopia lentis (CEL) patients and to evaluate the sensitivity and specificity of anterior segment parameters in distinguishing CEL from healthy controls. *Setting*. Zhongshan Ophthalmic Center, Guangzhou, China.

**Design:**

Cross-sectional study.

**Methods:**

35 CEL patients and 35 age- and sex-matched healthy controls were recruited. Axial length (AL) and anterior segment parameters including anterior chamber width (ACW), angle open distance (AOD), angle recess area (ARA), trabecular-iris space area (TISA), and trabecular-iris angle (TIA) were measured. All the above parameters and the ratio index of angle parameters, which was defined as the angle parameter value of the narrower side to that of the contralateral side, were compared between CEL and controls. Receiver operating characteristic (ROC) curves were also plotted to evaluate the diagnostic performance of anterior chamber angle parameters in CEL patients.

**Results:**

All angle parameters of the contralateral side to the dislocated lens side were significantly smaller than those of the dislocated lens side in CEL (all *P* < 0.05). For the diagnostic performance of anterior chamber angle parameters, the ratio index of TIAr500 combined with TIAr750 had the best diagnostic performance for CEL screening (AUC = 0.798), and TIAr500 of 0.887 and TIAr750 of 0.917 were detected to be the optimal cut-off points, representing a sensitivity of 89.8% and specificity of 68.7%.

**Conclusion:**

The contralateral side to the dislocated lens side in the CEL had a narrower anterior chamber angle. TIAr500 combined with TIAr750 is the optimal combination strategy for ectopia lentis screening.

## 1. Introduction

Congenital ectopia lentis (CEL) is a rare disease in which the lens dislocates from its normal position; it is a hereditary connective tissue disease [1]. CEL can not only lead to ocular symptoms such as severe refractive errors and amblyopia but also be associated with systemic diseases, such as such as Marfan's syndrome (MFS), homocystinuria, Weill–Marchesani syndrome, and sulfite oxidase deficiency syndrome [2].

For eyes with ectopia lentis, it has been reported that the dislocated lens can shallow the anterior chamber angle (ACA) and even cause acute secondary angle closure [3]. Zhang et al. [4] reported that CEL accounts for 2.4% of all causes of secondary glaucoma. However, little is known about the anterior segment characteristics of CEL patients, which hinders our understanding of the disease. While the slit lamp biomicroscope has been widely used for diagnosing ectopia lentis, this examination method may not detect early mild lesions without obvious signs; hence, objective and accurate diagnosis strategies for lens dislocation are needed.

In this study, we aimed to use the latest anterior segment swept-source optical coherence tomography (SS-OCT), a noncontact instrument with high-resolution imaging [5], to investigate the anterior segment parameters of patients with CEL and to evaluate the sensitivity and specificity of anterior segment parameters in distinguishing ectopia lentis from healthy controls.

## 2. Methods

This cross-sectional study was conducted in accordance with the tenets of the Declaration of Helsinki and was approved by the Institutional Review Board of Zhongshan Ophthalmic Center. The CEL patients and the healthy controls were consecutively recruited from January 2021 to August 2021 in Zhongshan Ophthalmic Center, Sun Yat-sen University, Guangzhou, China. The included CEL patients were diagnosed according to the Ghent-2 criteria with genetic testing [6]. Exclusion criteria were as follows: (1) patients with ocular surgery history; (2) with other ocular diseases such as corneal diseases which would affect the measurement of anterior segment parameters. Age- and sex-matched individuals without ocular disease other than refractive error were included as the healthy controls. Written informed consent was obtained from all participants before enrolling.

All participants underwent a standardized ophthalmic examination including slit-lamp biomicroscopy, best corrected visual acuity, intraocular pressure (IOP) with Goldmann applanation, AL with IOL Master700 (Zeiss, Jena, Germany), and anterior segment SS-OCT images with the Casia SS-1000 OCT (Tomey, Nagoya, Japan).

## 3. Swept-Source Anterior Segment Optical Coherence Tomography Examination

The Casia SS-1000OCT (Tomey, Nagoya, Japan) is a commercially available swept-source OCT system with a swept-source laser wavelength of 1310 nm, using a monochromatic, tunable, fast scanning laser source and a photodetector to detect wavelength-resolved interference signals. During the examination, all the participants were asked to fixate on an internal fixation target during the scan. All images were obtained in a dark environment by the same observer before the participants received any pupil dilation or constriction medications. For the patients with mild subluxation, we also performed SS-OCT scanning both under nonmydriatic pupil (to collect the angle parameters) and mydriatic pupil (to confirm the direction of lens dislocation). To avoid lid artifact, participants were instructed to pull down the lower lid against the lower orbital rim to expose the lower limbus while the technician elevated the upper lid against the upper orbital rim to expose the upper limbus. All images with lid or motion artifacts were excluded from the analysis.

**Figure 1:**
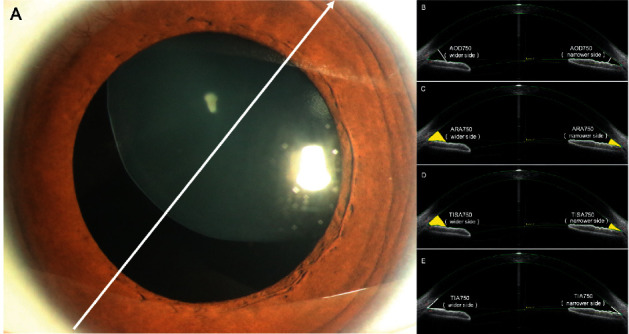
Diagram of the scan performed in CEL patients. (a) The scan was performed on the dislocated lens axis. (b–e) Different angle parameters of the dislocated lens side (wider side) and the contralateral side to the dislocated lens side (narrower side), and the ratio index of angle parameter was defined as the angle parameter value of the narrower side to that of the contralateral side.

Anterior segment parameters of different axes were obtained from each participant. For CEL patients, the scans were performed on the dislocated lens axis (Figure 1). For healthy controls, the scans were performed on the horizontal axis (0–180 degrees) and vertical axis (90–270 degrees). For each image, the scleral spur (SS) and angle recess (AR) were both marked by the SS-OCT system first and then manually corrected to complete anterior chamber measurements by the same experienced ophthalmologist (Liu ZZ). The scanned images were analyzed using custom software, and the obtained parameters included the following: anterior chamber dimension parameters (anterior chamber width (ACW)), angle parameters (angle opening distance (AOD), angle recess area (ARA), trabecular-iris space area (TISA), and trabecular-iris angle (TIA)). AOD, ARA, TISA, and TIA were all assessed at 250 *μ*m, 500 *μ*m, and 750 *μ*m from the scleral spur. All SS-OCT anterior chamber parameters are shown in Supplemental Figure 1. Of the above parameters, ACW was defined as the distance between the two scleral spurs. Angle opening distances at 250, 500, and 750 *μ*m (AOD250, AOD500, and AOD750) were defined as the distance between the posterior corneal surface and the anterior iris surface on a line perpendicular to the trabecular meshwork 250 *μ*m, 500 *μ*m, and 750 *μ*m from the scleral spur, respectively. Angle recess areas at 250, 500, and 750 *μ*m (ARA250, ARA500, and ARA750) were defined as the area of the angle recess bounded anteriorly by the AOD250, AOD500, and AOD750. Trabecular-iris space areas at 250, 500, and 750 *μ*m (TISA250, TISA500, and TISA750) were defined as the area bounded anteriorly by AOD250, AOD500, and AOD750 as determined posteriorly by a line drawn from the scleral spur vertical to the plane of the inner scleral wall to the iris, superiorly by the inner corneoscleral wall, and inferiorly by the iris surface. Trabecular-iris angles at 250, 500, and 750 *μ*m (TIA250, TIA500, and TIA750) were defined as an angle measured with the apex in the iris recess and the arms of the angle passing through a point on the trabecular meshwork 250 *μ*m, 500 *μ*m and 750 *μ*m from the scleral spur and the point on the iris perpendicularly.

For the CEL patients and healthy controls, the ratio index of angle parameters, defined as the angle parameter value of the narrower side to that of the contralateral side, was also introduced as AODr, ARAr, TISAr, and TIAr in this study. The angle parameters are shown in Figure 1.

## 4. Statistical Analysis

All statistical analysis was performed using Stata MP 15.1 (Stata Corp LP, College Station, Texas, USA). Mean values with 95% confidence intervals (95% CI) were provided for normally distributed data. All data were tested for normality. Normally distributed parameters were compared between the CEL and healthy controls using the student's *t* test, while the rank sum test was used for nonnormal distribution data. The receiver operating characteristics (ROC) curve was plotted to evaluate the diagnostic value of each SS-OCT parameter in the differential diagnosis of CEL patients from healthy controls. A value of *P* < 0.05 was considered statistically significant unless otherwise specified.

## 5. Results

A total of 35 eyes from 35 CEL patients were recruited, of which 18 were male (51.43%) and 17 were female (48.57%). Meanwhile, 35 eyes from age- and sex-matched healthy controls were recruited, of which 17 were male (48.57%) and 18 were female (51.43%). The mean age of the CEL patients was 13.57 ± 8.37 years, and the healthy control group age was 11.11 ± 2.99 years. The mean AL was 25.01 ± 2.76 mm in the CEL and 25.01 ± 2.76 mm in the healthy controls, with no significant difference between the two groups were detected. Demographics and ocular biometric characteristics of participants are shown in Table 1.

**Figure 2:**
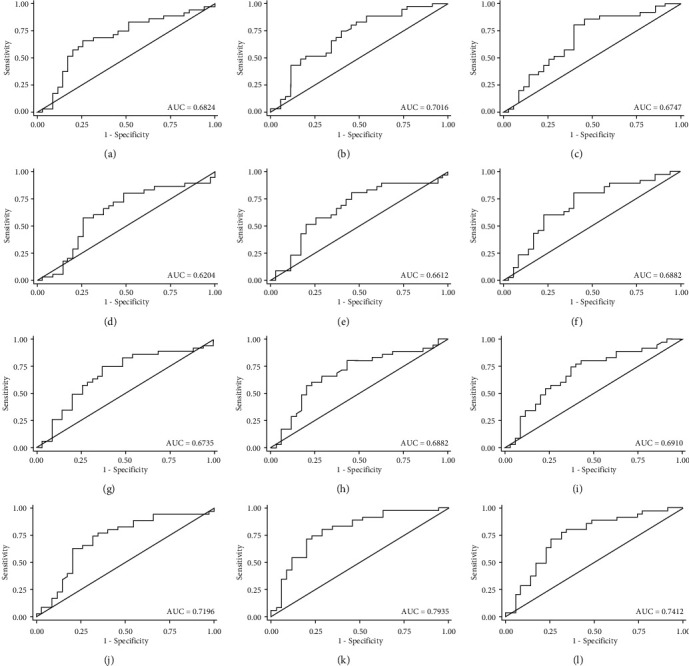
The receiving operating characteristics curve of single ratio index of angle parameter in differential diagnose of ectopia lentis. ((a) AODr250 (AUC = 0.682); (b) AODr500 (AUC = 0.701); (c) AODr750 (AUC = 0.675); (d) ARAr250 (AUC = 0.620); (e) ARAr500 (AUC = 0.661); (f) ARAr750 (AUC = 0.688); (g) TISAr250 (AUC = 0.674); (h) TISAr500 (AUC = 0.688); (i) TISAr750 (AUC = 0.691); (j) TIAr250 (AUC = 0.720); (k) TIAr500 (AUC = 0.794); (l) TIAr750 (AUC = 0.741).

**Figure 3:**
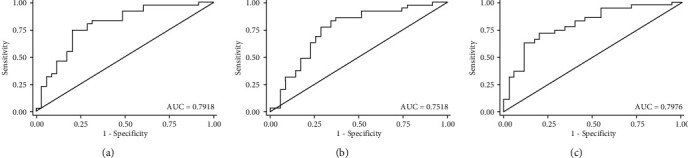
The receiving operating characteristics curve of combined ratio index of angle parameters in differential diagnose of ectopia lentis. ((a) TIAr250+TIAr500 (AUC = 0.792); (b) TIAr250+TIAr750 (AUC = 0.752); (c) TIAr500+TIAr750 (AUC = 0.798).

The angle parameters (AOD, ARA, TISA, and TIA) at 250 *μ*m, 500 *μ*m, 500 *μ*m, and 750 *μ*m of the dislocated lens side, the contralateral side to the dislocated lens side in the CEL patients, and those of the horizontal axis (0 degree and 180 degrees) and vertical axis (90 degree and 270 degrees) in the healthy controls are reported in Table 2. All angle parameters of the contralateral side to the dislocated lens side were significantly smaller than those of the dislocated lens side in the CEL patients and those of the horizontal axis (0–180 degrees) and vertical axis (90–270 degrees) in the healthy controls (all *P* < 0.05). As shown in Table 3, there were significant differences for all ratio indexes of angle parameters between the CEL patients and the healthy controls. To evaluate the sensitivity and specificity of the anterior segment parameters in distinguishing ectopia lentis from healthy controls, we calculated the area under the receiving operating characteristics curve (AUROC), which is presented in Figures 2 and 3. The ROC curve indicated that TIAr250, TIAr500, and TIAr750 had good discriminative performance (AUC = 0.720; AUC = 0.794; AUC = 0.741, respectively) for CEL. Meanwhile, the ratio index of TIAr500 combined with TIAr750 had the best diagnostic performance for CEL (AUC = 0.798); and TIAr500 of 0.887 and TIAr750 of 0.917 were found to be the optimal cut-offs.

Among the included CEL patients, FBN1 mutations account for more than 90% of the detected mutations. There was no statistical difference of anterior segment parameters among patients with different mutations.

## 6. Discussion

For CEL patients, the zonule of the lens is partially or completely slack or even broken, and the lens is often pushed forward to the iris which can change the anterior chamber angle structure. Until now, little is known about the characteristics of the anterior segment in CEL patients. Although changes in the anterior chamber depth can be easily detected in patients with severe lens dislocation, it is difficult to detect lens dislocation with traditional equipment such as a slit-lamp biomicroscope for patients with mild lens dislocation, and new detection strategies are needed for these patients.

At present, SS-OCT serves as a noncontact, easy-to-use, and quantitative evaluation of the anterior chamber device [7, 8] that utilizes much more data of the whole anterior chamber than can be obtained by the commonly used UBM. In addition, the noncontact nature of SS-OCT makes it more advantageous in pediatric patients with CEL. With the advancement of OCT technology, the current ultrahigh resolution of OCT can obtain very clear imaging of various structures in the anterior segment of the eye, and AS-OCT has been successfully used in the diagnosis of a variety of anterior segment diseases [9–11]. Ma et al. [12] reported that SS-OCT showed excellent diagnostic ability in distinguishing primary angle closure disease (PACD) from the healthy population and showed moderate diagnostic performance in distinguishing primary angle closure/primary angle closure glaucoma (PAC/PACG) from primary angle closure suspect (PACS). However, the use of SS-OCT in the diagnosis of CEL patients has not been reported by far.

In this study, we detected that all angle parameters of the contralateral side to the dislocated lens side in CEL patients were significantly smaller than those of the healthy controls (*P* < 0.05). Specifically, the angle parameters of the contralateral side to the dislocated lens side in the CEL patients were statistically smaller than those of the opposite side (*P* < 0.05). The explanation of our results may lie in the fact that the dislocated lens could cause mechanical compression of the ACA, hence affecting the angle parameters. Previous studies have reported that angle parameters were significantly associated with angle closure [13–16]. Previous reports by Henriquez et al. [17] identified that TISA500 < 0.009 was risk factor for developing phacomorphic angle closure, and narrower angle width was an independent predictive factor for the development of angle closure after ten years [18]. Additionally, the smaller AOD750 was also identified to be strongly associated with the development of incident angle closure in a previous study [19]. Due to the angle characteristics of the CEL patients, much more attention should be paid to the changes in angle parameters, and regular follow-up is recommended.

In this study, we also introduced a series ratio index of the angle parameters such as TIAr, AODr, ARAr, and TISAr. There was a significant difference between the CEL patients and the healthy controls for all angle parameter ratio indexes, of which the ratio indexes in CEL patients were smaller compared to the healthy controls. To explore the potential value of angle parameters in the differential diagnosis of lens dislocation, ROCs were plotted, and the results showed that TIAr had a higher power of discrimination compared with AODr, ARAr, and TISAr (all *P* < 0.05). The TIA is a parameter that represents the angle width, which has been commonly used in the evaluation of the ACA [20, 21]. In order to improve the diagnostic efficiency of the ratio parameters, TIAr250, TIAr500, and TIAr750 were combined, and the results showed that TIAr500 combined TIAr750 (AUC = 0.798) had a promising diagnostic performance for lens dislocation. This suggests that TIAr500 combined with TIAr750 could serve as a screening tool for lens dislocation.

There are several limitations to this study. First, not all the cross-sectional images were analyzed, and the accuracy may be affected to some extent. Second, the scleral spur and angle recess required manual labeling. Although the investigator in this study had been well trained before the study, bias may still exist. Finally, since our participants were all from a single eye center, the generalization of our conclusion may be limited and further studies are needed. However, the value of our study is in the use of advanced SS-AS-OCT technology to evaluate the anterior segment characteristics of CEL patients and investigate the possibility of utilizing anterior angle parameters to differentiate and diagnose ectopia lentis patients from healthy controls, which could be a significant contribution to disease management.

In conclusion, the ACA of the contralateral side to the dislocated lens in CEL patients was significantly narrower than that of the healthy controls. We recommend that clinicians remain attentive to these changes in values, and regular follow-up is recommended for these patients. For the differential diagnosis of ectopia lentis, TIAr500 combined with TIAr750 could serve as a screening tool in clinical practice.

## Figures and Tables

**Table 1:** Demographics and ocular biometric characteristics of participants.

	CEL (*n* = 35)	Healthy controls (*n* = 35)
Age, years	13.571 ± 8.374	11.114 ± 2.988
Sex (male/female)	18/17	17/18
Eyes (right/left)	21/14	35/0
AL, mm	25.008 ± 2.764	24.651 ± 0.969
ACW, mm	11.966 ± 0.470	11.963 ± 0.357

AL = axial length; ACW = anterior chamber width.

**Table 2:** Comparison of angle parameters in different direction in CEL patients and healthy controls.

Angle parameters	CEL		Healthy controls			
	Contralateral side to dislocated lens side	Dislocated lens side (Mean ± SD, *P*^*∗*^)	0° (Mean ± SD, *P*^*∗*^)	90° (Mean ± SD, *P*^*∗*^)	180° (Mean ± SD, *P*^*∗*^)	270° (Mean ± SD, *P*^*∗*^)
AOD250, mm	0.27 ± 0.13	0.46 ± 0.16 <0.001	0.51 ± 0.13 <0.001	0.45 ± 0.15 <0.001	0.57 ± 0.18 <0.001	0.46 ± 0.14 <0.001
AOD500, mm	0.37 ± 0.15	0.63 ± 0.19 <0.001	0.74 ± 0.16 <0.001	0.67 ± 0.19 <0.001	0.82 ± 0.23 <0.001	0.66 ± 0.16 <0.001
AOD750, mm	0.51 ± 0.19	0.84 ± 0.22 <0.001	0.98 ± 0.18 <0.001	0.89 ± 0.21 <0.001	1.10 ± 0.25 <0.001	0.89 ± 0.22 <0.001
ARA250, mm^2^	0.10 ± 0.06	0.17 ± 0.09 <0.001	0.18 ± 0.05 <0.001	0.15 ± 0.07 <0.001	0.19 ± 0.06 <0.001	0.16 ± 0.06 <0.001
ARA500, mm^2^	0.17 ± 0.08	0.29 ± 0.13 <0.001	0.33 ± 0.09 <0.001	0.29 ± 0.10 <0.001	0.35 ± 0.11 <0.001	0.29 ± 0.10 <0.001
ARA750, mm^2^	0.28 ± 0.12	0.48 ± 0.18 <0.001	0.55 ± 0.13 <0.001	0.48 ± 0.14 <0.001	0.59 ± 0.17 <0.001	0.48 ± 0.14 <0.001
TISA250, mm^2^	0.057 ± 0.03	0.10 ± 0.04 <0.001	0.11 ± 0.03 <0.001	0.09 ± 0.04 <0.001	0.12 ± 0.04 <0.001	0.10 ± 0.03 <0.001
TISA500, mm^2^	0.14 ± 0.06	0.24 ± 0.09 <0.001	0.27 ± 0.07 <0.001	0.24 ± 0.08 <0.001	0.30 ± 0.09 <0.001	0.24 ± 0.07 <0.001
TISA750, mm^2^	0.25 ± 0.10	0.43 ± 0.14 <0.001	0.50 ± 0.11 <0.001	0.44 ± 0.12 <0.001	0.54 ± 0.15 <0.001	0.44 ± 0.11 <0.001
TIA250,°	33.51 ± 12.61	44.41 ± 8.40 <0.001	49.70 ± 11.36 <0.001	44.28 ± 12.11 <0.001	52.42 ± 11.59 <0.001	45.65 ± 11.61 <0.001
TIA500°	29.70 ± 8.13	41.45 ± 7.09 <0.001	47.29 ± 9.13 <0.001	43.37 ± 9.68 <0.001	49.92 ± 9.16 <0.001	43.41 ± 9.64 <0.001
TIA750°	28.99 ± 7.99	40.84 ± 6.99 <0.001	46.37 ± 7.19 <0.001	43.14 ± 7.95 <0.001	49.65 ± 7.57 <0.001	42.93 ± 8.49 <0.001

AOD, angle open distance; ARA, angle recess area; TISA, trabecular-iris space area; TIA, trabecular-iris angle; ^*∗*^*t*-test for comparison of means with angle parameters of contralateral side to dislocated lens side in CEL patients.

**Table 3:** Comparison of the ratio index of different angle parameters in CEL patients with healthy controls (mean ± SD).

The ratio index of angle parameters	CEL	Healthy controls	*P*value
AODr250	0.65 ± 0.47	1.02 ± 0.31	<0.001
AODr500	0.63 ± 0.31	1.03 ± 0.21	<0.001
AODr750	0.65 ± 0.35	1.04 ± 0.20	<0.001
ARAr250	0.64 ± 0.48	1.02 ± 0.44	<0.05
ARAr500	0.63 ± 0.40	1.01 ± 0.26	<0.001
ARAr750	0.64 ± 0.36	1.02 ± 0.22	<0.001
TISAr250	0.62 ± 0.42	1.02 ± 0.25	<0.001
TISAr500	0.63 ± 0.37	1.02 ± 0.21	<0.001
TISAr750	0.64 ± 0.35	1.03 ± 0.20	<0.001
TIAr250	0.79 ± 0.41	0.99 ± 0.14	<0.05
TIAr500	0.73 ± 0.26	1.02 ± 0.15	<0.001
TIAr750	0.72 ± 0.27	1.03 ± 0.13	<0.001

^*∗*^The ratio index of angle parameter was defined as the ratio of the angle parameter value of the narrower side to that of the contralateral side.

## Data Availability

The data used to support the findings of this study are included within the article.

## Additional Points

*What Was Known.* (i) For eyes with ectopia lentis, the dislocated lens can squeeze the anterior chamber angle and even cause acute secondary angle closure, but little is known about the anterior segment characteristics of CEL patients. *What This Paper Adds*. (i) The anterior chamber angle of the contralateral side to the dislocated lens in congenital ectopia lentis was significantly narrower than that of the healthy controls. (ii) TIAr500 combined with TIAr750 could serve as a screening tool in differential diagnosis of ectopia lentis form healthy population.

## Supplementary Materials

Supplementary Materials Supplemental Figure 1: diagram of anterior chamber angle parameters in SS-OCT (a: anterior chamber width (ACW); b: angle opening distance (AOD) at 250, 500, and 750 *μ*m; c–e: angle recess area (ARA) at 250, 500, and 750 *μ*m; f–h: trabecular-iris space area (TISA) at 250, 500, and 750 *μ*m; i–k: trabecular-iris angle (TIA) at 250, 500, and 750 *μ*m).
