# Supplementary figures and images for: Characteristics of Anterior Segment in Congenital Ectopia Lentis: An SS-OCT Study
Source: J Ophthalmol. 2022 Jun 6;2022:6128832. doi: 10.1155/2022/6128832 (PMC9192328; doi:10.1155/2022/6128832)

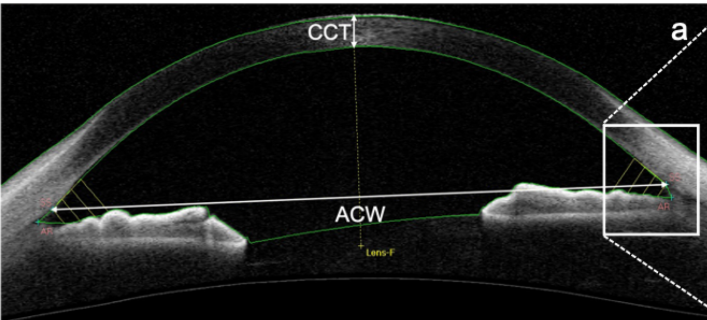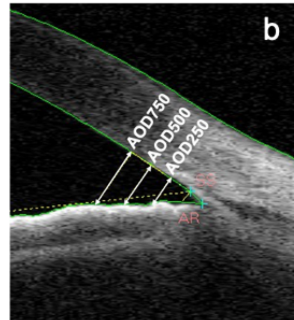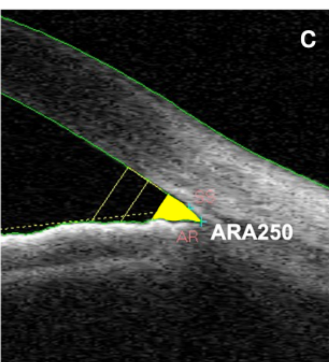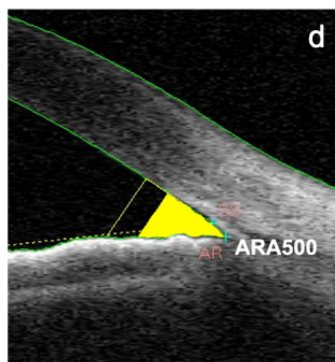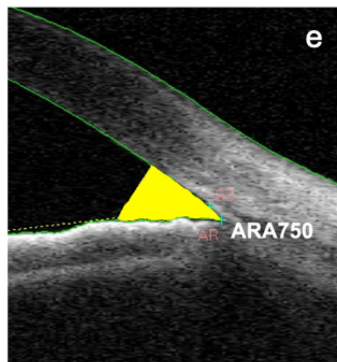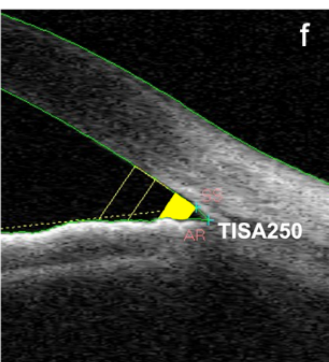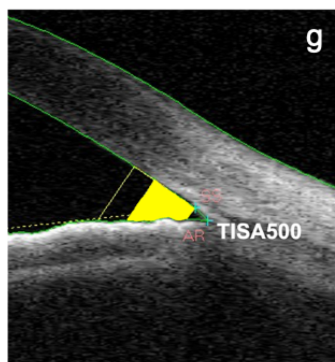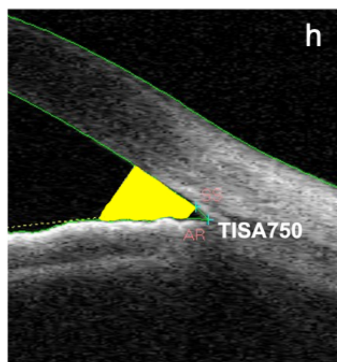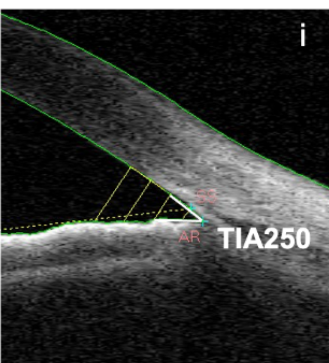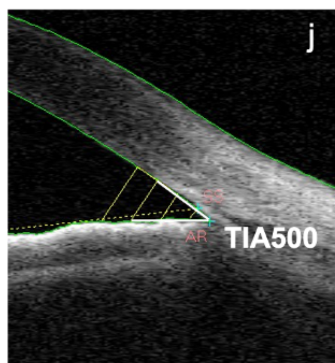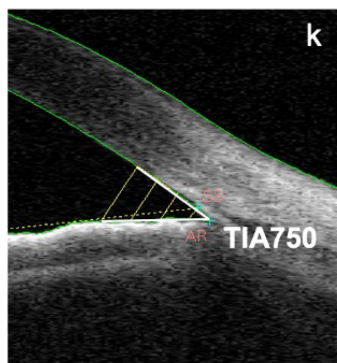

Supplement: Supplementary Materials — Supplemental Figure 1: diagram of anterior chamber angle parameters in SS-OCT (a: anterior chamber width (ACW); b: angle opening distance (AOD) at 250, 500, and 750 μm; c–e: angle recess area (ARA) at 250, 500, and 750 μm; f–h: trabecular-iris space area (TISA) at 250, 500, and 750 μm; i–k: trabecular-iris angle (TIA) at 250, 500, and 750 μm). [file 6128832.f1.pdf]
